# Successful Induction of Specific Immunological Tolerance by Combined Kidney and Hematopoietic Stem Cell Transplantation in HLA-Identical Siblings

**DOI:** 10.3389/fimmu.2022.796456

**Published:** 2022-01-31

**Authors:** Thomas Fehr, Kerstin Hübel, Olivier de Rougemont, Irene Abela, Ariana Gaspert, Tayfun Güngör, Mathias Hauri, Birgit Helmchen, Claudia Linsenmeier, Thomas Müller, Jakob Nilsson, Oliver Riesterer, John D. Scandling, Urs Schanz, Pietro E. Cippà

**Affiliations:** ^1^ Division of Nephrology, University Hospital Zurich, Zurich, Switzerland; ^2^ Department of Internal Medicine, Cantonal Hospital Graubuenden, Chur, Switzerland; ^3^ Department of Surgery and Transplantation, University Hospital Zurich, Zurich, Switzerland; ^4^ Institute of Medical Virology, University of Zurich, Zurich, Switzerland; ^5^ Department of Pathology, University Hospital Zurich, Zurich, Switzerland; ^6^ Division of Stem Cell Transplantation, University Children’s Hospital Zurich – Eleonore Foundation & Children`s Research Center (CRC), Zurich, Switzerland; ^7^ Department of Radiation Oncology, University Hospital Zurich, Zurich, Switzerland; ^8^ Laboratory for Transplantation Immunology, University Hospital Zurich, Zurich, Switzerland; ^9^ Division of Nephrology, Stanford University School of Medicine, Stanford, CA, United States; ^10^ Department of Medical Oncology and Hematology, University Hospital Zurich, Zurich, Switzerland; ^11^ Division of Nephrology, Ente Ospedaliero Cantonale, Lugano, Switzerland

**Keywords:** chimerism, hematopoietic stem cell transplantation (HSCT), tolerance, kidney transplantation, immunocompetence, COVID - 19

## Abstract

Induction of immunological tolerance has been the holy grail of transplantation immunology for decades. The only successful approach to achieve it in patients has been a combined kidney and hematopoietic stem cell transplantation from an HLA-matched or -mismatched living donor. Here, we report the first three patients in Europe included in a clinical trial aiming at the induction of tolerance by mixed lymphohematopoietic chimerism after kidney transplantation. Two female and one male patient were transplanted with a kidney and peripherally mobilized hematopoietic stem cells from their HLA-identical sibling donor. The protocol followed previous studies at Stanford University: kidney transplantation was performed on day 0 including induction with anti-thymocyte globulin followed by conditioning with 10x 1.2 Gy total lymphoid irradiation and the transfusion of CD34+ cells together with a body weight-adjusted dose of donor T cells on day 11. Immunosuppression consisted of cyclosporine A and steroids for 10 days, cyclosporine A and mycophenolate mofetil for 1 month, and then cyclosporine A monotherapy with tapering over 9–20 months. The 3 patients have been off immunosuppression for 4 years, 19 months and 8 months, respectively. No rejection or graft-versus-host disease occurred. Hematological donor chimerism was stable in the first, but slowly declining in the other two patients. A molecular microscope analysis in patient 2 revealed the genetic profile of a normal kidney. No relevant infections were observed, and the quality of life in all three patients is excellent. During the SARS-CoV-2 pandemic, all three patients were vaccinated with the mRNA vaccine BNT162b2 (Comirnaty^®^), and they showed excellent humoral and in 2 out 3 patients also cellular SARS-CoV-2-specific immunity. Thus, combined kidney and hematopoietic stem cell transplantation is a feasible and successful approach to induce specific immunological tolerance in the setting of HLA-matched sibling living kidney donation while maintaining immune responsiveness to an mRNA vaccine (ClinicalTrials.gov: NCT00365846).

## 1 Introduction

Kidney transplantation is the primary option for treatment of end stage renal failure in patients without contraindication for life-long immunosuppression. Since kidney allograft survival early after transplantation has substantially improved, the focus of research and clinical care has turned to improving long-term patient and allograft survival (1). Under long-term immunosuppression, patient survival is shortened due to neoplastic, infectious, and cardiovascular complications, whereas allograft survival is limited due to chronic rejection, drug toxicity, infections (such as BK virus nephropathy) or unspecific allograft injury and fibrosis. All these complications could either be controlled, reduced or completely avoided if successful immunologic tolerance was induced (2).

In pre-clinical models, various approaches have been successfully tested to induce tolerance to fully mismatched allografts, including co-stimulation blockade, donor-specific transfusion, or transfer of different types of regulatory cells (such as regulatory T cells, macrophages or tolerogenic dendritic cells). However, the only approach that was successfully translated into non-human primate models and clinical studies relies on hematopoietic stem cell transplantation (HSCT) leading to mixed lymphohematopoietic chimerism and transplantation of a kidney from the same donor (3).

Three groups in the United States have independently developed protocols to achieve this goal, using various conditioning regimens, stem cell preparations and timings (pre- versus post-kidney transplant conditioning) (4–6). An overview of these approaches is shown in **Supplementary Table 1**. Only the group in Stanford established a protocol that uses post-kidney transplant conditioning and HSCT, which theoretically allows to translate this approach also to deceased donor transplantation. Therefore, we decided to implement a similar protocol for the first trial of combined kidney transplantation and HSCT in Europe. Here we report the results of the first three patients enrolled in this trial (swisstolerance.CH).

## 2 Methods

### 2.1 Trial Design

This is an open-label feasibility study of combined HLA-matched (10/10; Loci A/B/C/DR/DQ) sibling kidney and hematopoietic stem cell transplantation to induce donor-specific immunological tolerance to the kidney allograft.

The *primary endpoint* of the study was renal allograft acceptance and ability to discontinue immunosuppressive therapy at 1 year.

*Secondary endpoints* were engraftment of donor hematopoietic stem cells (chimerism) measured at 6 months, absence of graft-versus-host disease (GVHD) after 6 and 12 months, absence of renal allograft rejection at 6 and 12 months, T cell recovery and immune reconstitution, absence of opportunistic infections (immune competence) and quality of life.

*Chimerism* is defined as evidence of donor-derived hematopoietic cells in peripheral blood measured by Variable number tandem repeats (VNTR).

### 2.2 Patient Population

#### 2.2.1 Inclusion Criteria

All patients aged 18-70 with end-stage renal failure under evaluation for kidney transplantation at the University Hospital Zurich were considered for this clinical trial. Subjects had to have an HLA-matched sibling donor 18-70 years of age and be able to understand and provide informed consent.

#### 2.2.2 Exclusion Criteria

The following exclusion criteria were applied:

- Evidence of uncontrolled active infection (including replicating HIV, HCV and HBV), serologic positivity to HIV
- Contraindication to therapy with any one of the proposed agents
- Women of childbearing age in whom adequate contraception could not be maintained, pregnant women or nursing mothers.
- Malignancy within the past two years, for which waiting time for transplantation is required by Israel Penn Registry consult, thereby excluding non-melanoma skin cancer and carcinoma *in situ* of the cervix.
- Relevant liver, cardiac or pulmonary disease
- ABO blood group incompatibility in the host-vs-graft direction (major incompatibility)
- Panel reactivity antibody >20%.
- Very high risk of primary kidney disease recurrence (mainly focal segmental glomerulosclerosis or atypical hemolytic uremic syndrome)

#### 2.2.3 Patient Recruitment

The kidney transplant center in Zurich performs approximately 90 kidney transplants per year, among those around 25 living donations. Every kidney transplant candidate is systematically evaluated for a potential living donation. If a candidate had a potential sibling donor, they were introduced to the concept and the protocol of the swisstolerance.CH trial. Between 2016 until 2020, eight HLA-identical sibling pairs were evaluated, and 3 were included into this pilot trial. The other five pairs were excluded either due to ABO incompatibility, or the donor had contraindications to living donation. Among those, three pairs were transplanted regularly outside the protocol.

### 2.3 Study Protocol

#### 2.3.1 Interventions Before Transplantation

Donor and recipients were screened according to the established internal guidelines for living donor kidney and HSCT of the Transplantation Center of the University Hospital Zurich. For the planning of the total lymphoid irradiation (TLI) a planning CT was performed 2-4 weeks before transplantation and repeated on the day after kidney transplantation to shield the transplanted kidney.

Donor-derived hematopoietic progenitor cells were isolated from peripheral leukocytes after apheresis by positive selection (CD34+ cells) *via* magnetic cell sorting (CliniMACS, Miltenyi^®^, Germany) according to SOPs of the certified Stem cell laboratory of the University Children’s Hospital Zurich. CD34-negative cells were analyzed by flow cytometry to determine the number of CD3-positive cells (for T cell add-back). CD34+ cells and flow through cells were frozen in liquid nitrogen until use.

#### 2.3.2 Kidney Transplantation (Day -11)

**Figure 1:**
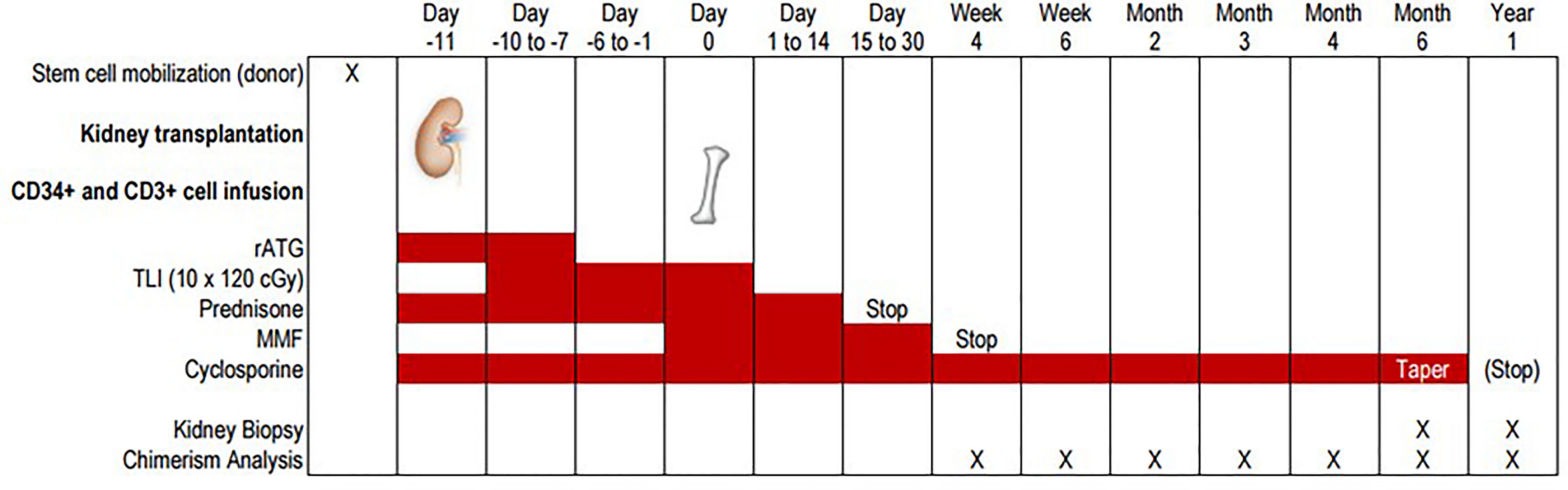
Overview over the trial protocol. Schematic overview over the trial protocol showing the timing of kidney and hematopoietic stem cell transplantation, immunosuppressive medication and monitoring with peripheral blood chimerism analyses and allograft biopsies.

Living kidney donation (laparoscopic approach) and transplantation were performed according to standard procedures. Immunosuppression in the first weeks after kidney transplantation included prednisone, mycophenolate mofetil and cyclosporine A (details of dosing: see 3.3.4). In addition, rabbit anti-thymocyte globulin (ATG, Thymoglobulin^®^) at a dose of 1.5 mg per kg body weight was applied from day -11 to day -7 (**Figure 1**).

#### 2.3.3 Hematopoietic Stem Cell Transplantation (HSCT, Day 0)

The conditioning regimen consisted of total lymphoid irradiation (10 daily doses of 120 cGy = total dose 12 Gy) each to the supradiaphragmatic lymph nodes, thymus, subdiaphragmatic lymph nodes and spleen. The treatment started 1 day after kidney transplantation (d-10).

On day 0, the isolated CD34+ hematopoietic progenitor cells (≥4x10^6^ cells/kg of the recipient`s body weight) were thawed an infused together with 1x10^6^ CD3+ T cells/kg from the CD34- fraction to promote the engraftment of hematopoietic progenitor cells (T cell add-back)

#### 2.3.4 Immunosuppression and Anti-Microbial Prophylaxis

Immunosuppression post-transplant was guided as follows:

- Methylprednisolone/Prednisone: steroids were rapidly tapered during the first days after transplantation. All patients were off steroids 14 days after kidney transplantation.
- Mycophenolate mofetil: 2 g per day (in 2 doses), started at day 0 (4 to 6 hours after HSCT) and discontinued 1 month after HSCT.
- Cyclosporine A: first 6 months whole blood through level (C0) 250-300 µg/L, after 6 months cyclosporine was tapered and discontinued if the following criteria were fulfilled:
- Sustained chimerism for at least 180 days, no clinical signs of rejection, protocol biopsy showing no evidence of acute or chronic rejection, no clinical signs of GvHD.
- Anti-microbial prophylaxis was performed as follows:
- Amoxicillin/clavulanic acid 2.2 g preoperatively;
- Sulfamethoxazole/Trimethoprim 3x/week for 6 months;
- Valganciclovir: a) low risk (D-R-) – no prophylaxis; b) intermediate risk (R+) – prophylaxis with valganciclovir 450 mg once daily, starting one month post-kidney transplant; c) high risk (D+R-) – prophylaxis with valganciclovir 450 mg once daily, starting immediately after kidney transplant.

#### 2.3.5 Post-Transplant Monitoring

Standard follow-up procedures for living kidney donors and recipients as established in the transplant center in Zurich were applied. In addition, during immunosuppression tapering and in the first months off immunosuppression renal function was weekly monitored for an early detection of rejection episodes. GVHD was monitored clinically at each regular visit as well as by measurement of liver function tests.

Donor chimerism level in peripheral blood was regularly assessed. Immune reconstitution was analyzed by flow cytometry of peripheral blood leukocytes.

Kidney allograft biopsy was performed at 6 months post-transplant and immediately before full withdrawal of cyclosporine A.

#### 2.3.6 Assessment of SARS-CoV-2-Specific Immunity

During the COVID-19 pandemic, all three trial patients were vaccinated against SARS-CoV-2 with the mRNA vaccine BNT162b2 (Comirnaty^®^, Pfizer/BioNTech), and the SARS-CoV-2-specific antibody and T cell responses were assessed several months after vaccination.

The SARS-CoV-2 antibody response was assessed by the commercially available ELISA assay (Elecsys^®^, Roche). To assess the neutralizing capacity of these antibodies, an additional assay developed by the Institute of Medical Virology (IMV, University of Zurich) was used (ABCORA®).

The SARS-CoV-2 T cell response was assessed with an *in vitro* T cell stimulation assay established in the laboratories of the Division of Clinical Immunology (University Hospital of Zurich). As a positive control, the T cell responses against a mitogen (Concanavalin A) and a bacterial superantigen (Staphylococcus aureus SEA/SEB) were evaluated. To assess the specific anti-viral T cell response, the SARS-CoV-2 spike protein and a CMV protein were used as antigens.

### 2.4 Ethical Approval and Trial Registration

This pilot trial was approved by the Ethical Committee for clinical research of the Canton of Zurich (KEK_ZH, application No 2013-0603). The trial is registered at ClinicalTrials.gov (Identifier NCT02176434).

## 3 Results

### 3.1 Individual Patients

#### 3.3.1 Patient 1

Our first patient was a 57-year-old Caucasian woman with end stage renal disease due to a glomerulopathy, that could not be specified at the time of diagnosis (**Table 1**). She received a preemptive kidney transplant from her 53-year-old HLA-identical brother. Immunosuppression followed the standard trial protocol (including ATG, cyclosporine, mycophenolate mofetil and steroids) during the first month, which was then continued with cyclosporine A monotherapy starting at month 2. Two kidney biopsies were performed according to protocol, the first at month 6, the second before discontinuation of cyclosporine at months 11 (**Supplementary Table 1**). Cyclosporine was gradually tapered after the first biopsy which did not show any signs of rejection, and it was discontinued after the second biopsy eleven months after transplantation (**Supplementary Figure 1**), still without signs of rejection. Whole blood donor chimerism at that time was around 50%.

**Table 1:** Patient and transplant characteristics.

Patient No		Patient 1	Patient 2	Patient 3
**Recipient**	Age, sex	57, F	61, F	49, M
	Renal disease	GN, unknown	GN, unknown	ADPKD
	Other diseases	M. Meniere	Breast cancer (9y before transplantation) Osteoporosis Neuroborreliosis	Nephrolithiasis
	Dialysis	None, preemptive transplant	Peritoneal dialysis (33 months)	Peritoneal dialysis (4 months)
	Transplant No	First	First	First
**Donor**	Age, sex	53, M	54, F	46, F
**HLA typing**	HLA class I	A1, A2; B8; B64(16); Cw7, Cw8	A2, -; B7, B62(15); Cw9; Cw10	A1, A3; B7, B8; Cw7, -
	HLA class II	DR17(3), DR7; DR52, DR53; DQ2, -; DP1, DP4	DR15(2); DR 13(6); DR51, DR52; DQ6(1), -; DP2, -	DR17(3); DR12(5); DR52, -; DQ2, DQ7; DP4, -
**HSCT**	No of CD34+ cells	8.78x10^6^/kg BW CD34+	5.61x10^6^/kg BW CD34+	12.3x10^6/^kg BW CD34+
	No of CD3+ cells (T cell add-back)	1x 10^6/^kg BW CD3+	1x 10^6/^kg BW CD 3+	1x10^6/^kg BW CD3+

ADPKD, adult polycystic kidney disease; BW, body weight; F, female; GN, glomerulonephritis; HSCT, hematopoietic stem cell transplantation; M, male; No, number.

The patient was never re-hospitalized after transplantation. Her first year was characterized by only few medical problems. She developed a calcineurin inhibitor pain syndrome (CIPS) with typical features on bone scintigraphy, 3 months after transplantation. It was well controlled with analgesics and disappeared when cyclosporine A was reduced and then stopped according to the trial protocol. One urinary tract infection, treated with antibiotics, occurred ten months after transplantation. Furthermore, asymptomatic low-level cytomegalovirus (CMV) reactivation was observed, which did not require treatment (maximum titer: 600 IU/mL). The patient returned to work two and a half months after transplantation.

**Figure 2:**
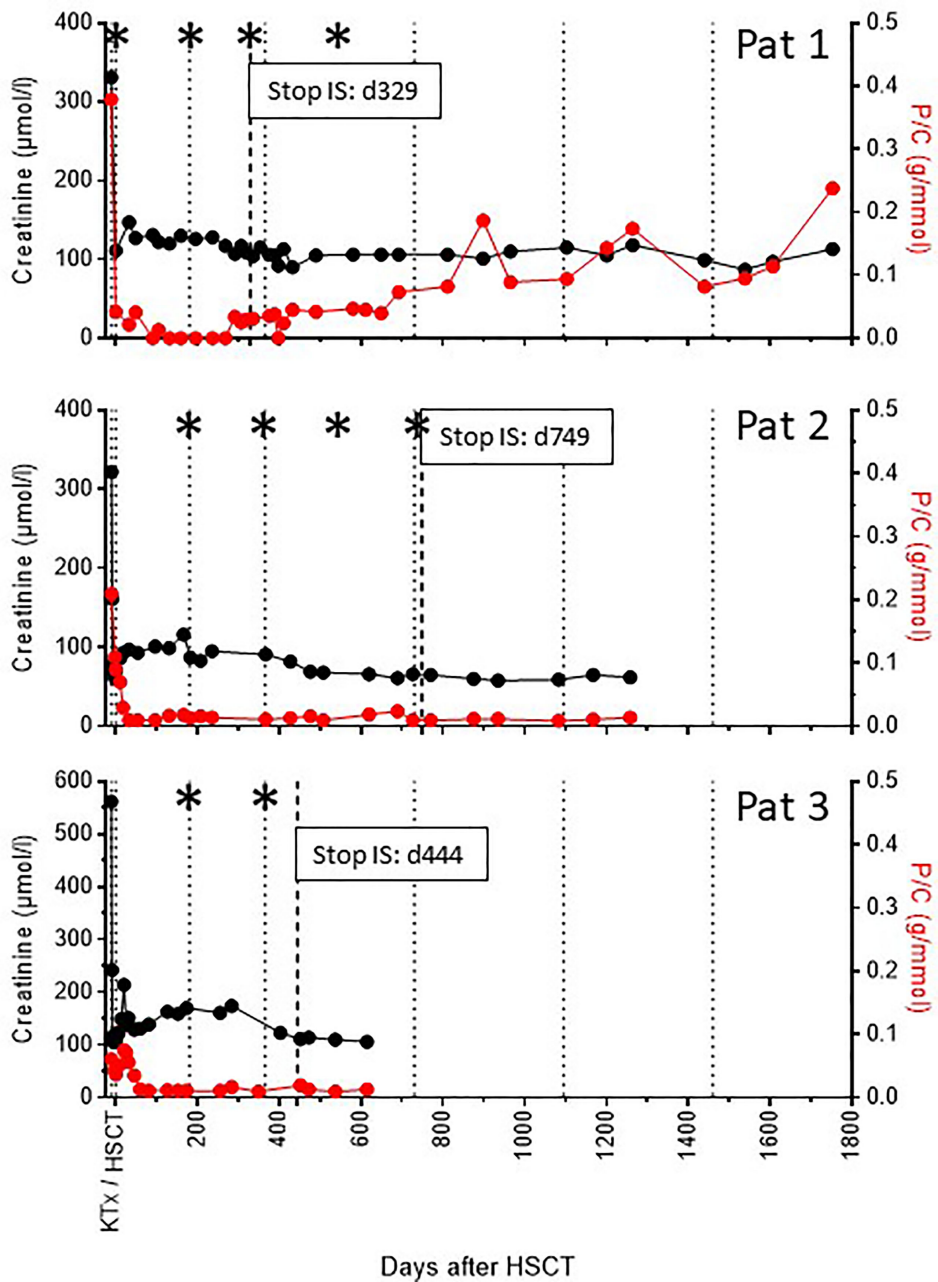
Synopsis of renal function and proteinuria over time. The course of allograft function (serum creatinine, black dots) and proteinuria (protein/creatinine ratio, red dots) over time is shown for all three recipients. Dotted lines indicate time intervals of 6 and 12 months post-transplant and yearly thereafter. The hatched line indicates the time point of stop of all immunosuppressive treatment (IS). Asterisks indicate time points of allograft biopsies (see also **Supplementary Table 1**).

Around one-year post-transplant, a low-level albuminuria was observed, which increased to about 1g/d by the end of the second year (**Figure 2**). The third kidney biopsy was performed 18 months post-transplant and revealed a primary glomerulonephritis (recurrent or *de novo*), which could not be further classified. The patient was treated for 10 weeks with mycophenolate mofetil, which however had no effect on proteinuria and was stopped due to gastrointestinal side effects. The patient was then switched to aliskiren (7).

The patient is now in her fifth year after transplantation with stable kidney function and albuminuria around 2 g/d.

#### 3.3.2 Patient 2

The second patient was a 61-year-old Caucasian woman with end stage renal disease due to a not further specified glomerulopathy. She was on peritoneal dialysis for 2 years and nine months before she received the kidney of her 54-year-old HLA-identical sister. Immunosuppression followed the standard trial protocol and was continued with cyclosporine A monotherapy starting at month 2. The donor chimerism level achieved was low. Therefore, we maintained the cyclosporine A whole blood through levels between 200-250 µg/L for 12 months (**Supplementary Figure 1**).

The first biopsy after 6 months showed very few lymphocytes in the peritubular capillaries (not diagnostic for peritubular capillaritis). We started cyclosporine A tapering after a normal second allograft biopsy one year after transplantation. Due to a very low chimerism level, tapering was performed very slowly. Cyclosporine A was finally stopped two years after transplantation (**Supplementary Figure 1**) and with a normal biopsy at that time point. The patient is now 3 years post-transplant and nineteen months without immunosuppressive therapy with stable kidney function and no proteinuria.

Since the transplantation, the patient has never been hospitalized. The only major complication was a Helicobacter-negative gastric ulcer, which was successfully treated by a proton pump inhibitor.

#### 3.3.3 Patient 3

The third patient was a 49-year-old Caucasian male with end stage renal disease due to adult polycystic kidney disease. He was on peritoneal dialysis for three and a half months before receiving a living donor kidney from his 46-year-old HLA-identical sister. Immunosuppression followed the standard trial protocol and was continued with cyclosporine monotherapy starting at month two. Early post-transplant, the patient had to be hospitalized for fenestration of a lymphocele.

The first allograft biopsy 6 months post-transplant did not show any signs of rejection (**Supplementary Table 2**). However, because of a rapid decline of donor chimerism, we maintained cyclosporine A whole blood though levels at 200-250 µg/L until months 9 and started tapering only thereafter. The second biopsy after 12 months during cyclosporine A tapering showed a BK-polyomavirus nephropathy. At this time point BK viremia was detected at very low level of about 975 IU/mL. Cyclosporine tapering was therefore continued, and since month 14 after transplantation the patient is without any immunosuppression (**Supplementary Figure 1**).

The patient is now 2 years post-transplant and 10 months without immunosuppressive therapy with stable kidney function, no signs of proteinuria (**Figure 2**), and no BK viremia. He returned to work 10 weeks post-transplant.

### 3.4 Synopsis of Renal and Hematologic Outcome

#### 3.4.1 Renal Outcome

All three patients achieved an immediate and excellent allograft function until the last follow-up 5 years, 3.5 years, and 1.7 years post-transplant (**Figure 2**). Proteinuria is normal in two of the patients, whereas patient 1 developed albuminuria up to 2g/d due to a glomerulonephritis (*de novo* or recurrent).

In a total of 9 allograft biopsies no signs of rejection were seen (**Supplementary Table 2**). In patient 3, the 12 months biopsy surprisingly showed a BK polyomavirus nephropathy. A low-level BK viremia was also found, which however immediately disappeared with stop of immunosuppression 14 months post-transplant. Kidney function remained stable over time (**Figure 2**).

**Figure 3:**
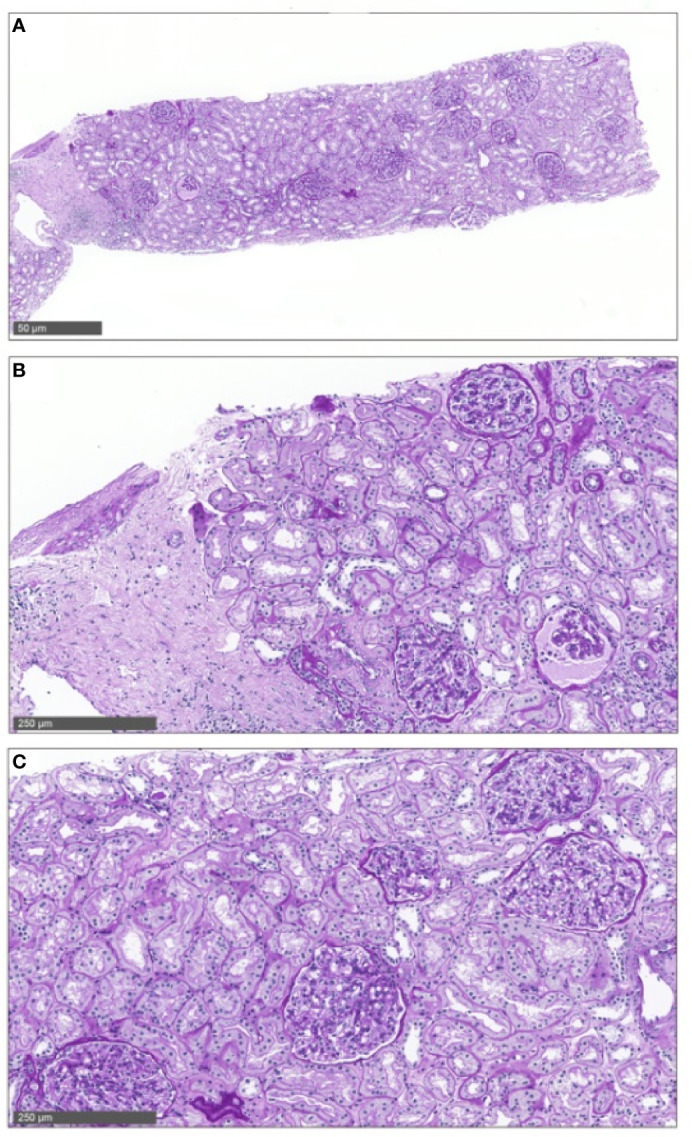
Allograft biopsy no 3 in patient 2. Allograft biopsy of patient 2 18 months post-transplant showing minimal glomerular alterations without signs of acute rejection. This biopsy was taken under minimal immunosuppressive therapy (cyclosporine A level at 13 ug/L), at the same time as the molecular microscope analysis shown in **Figure 4**.

**Figure 4:**
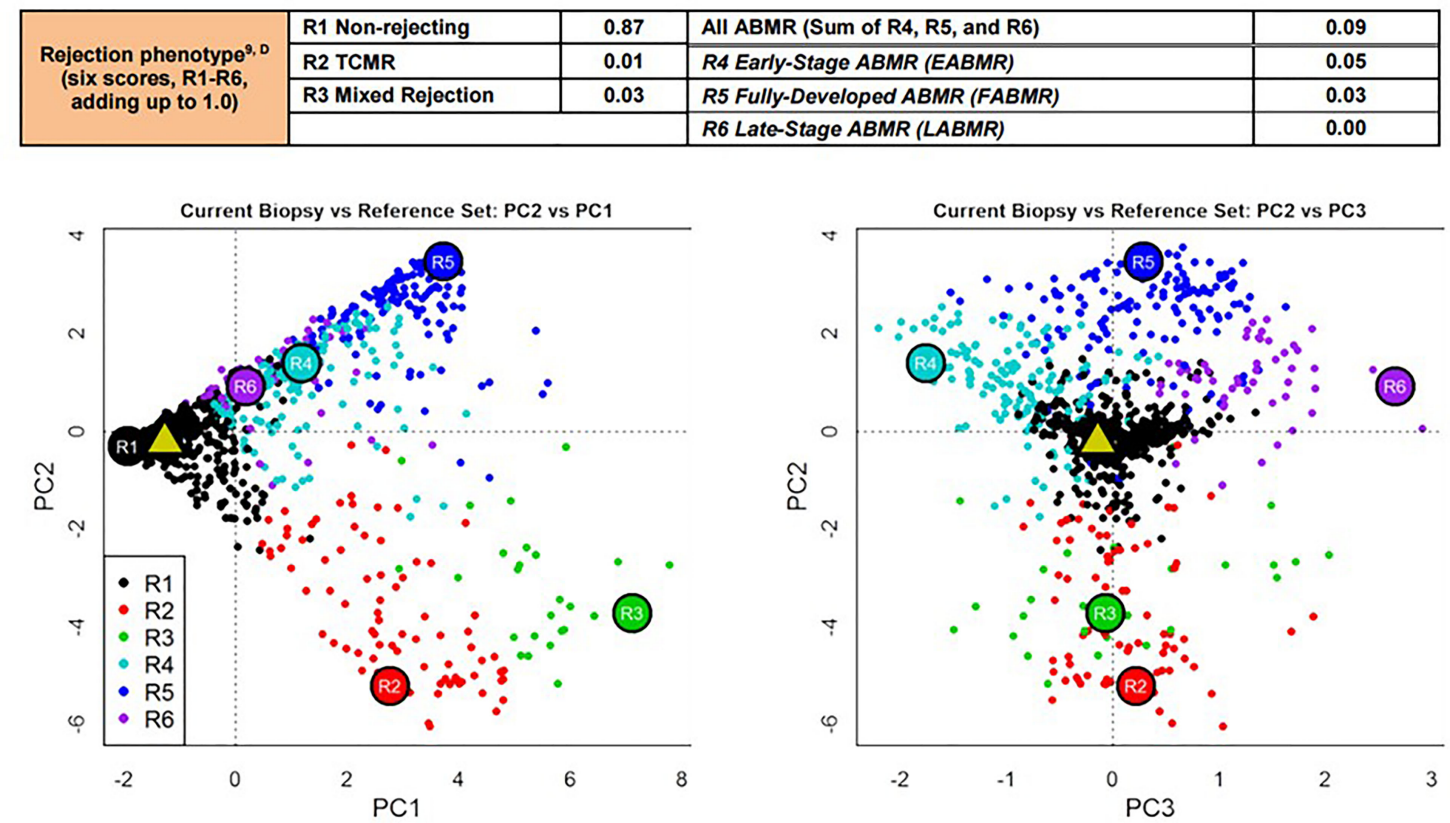
Molecular microscope analysis of allograft biopsy no 3 in patient 2. In this analysis, an mRNA microarray of 60 genes was performed to arrive at a molecular diagnosis of T-cell-mediated, antibody-mediated or mixed rejection. This analysis was performed 18 months post-transplant under very low levels of cyclosporine monotherapy (trough level of 13 µg/L, **Supplementary Figure 1**) and showed a completely normal gene expression pattern as seen in normal kidneys from living donors (black dots are normal kidney, the green triangle represents our patient).

In patient 2, the biopsy 18 months post-transplant showing minimal glomerular alterations (**Figure 3**) was analyzed in addition with the molecular microscope technology (MMDx), including an mRNA microarray of 60 genes, as previously described (8). This analysis showed a completely normal gene expression as seen in normal kidneys from living donors (**Figure 4**).

#### 3.4.2 Hematological Outcome

Allogeneic HLA-identical transplantation of peripherally mobilized hematopoietic stem cells was successfully performed in all three recipients without any transplant-related complications. Donor 2 did not mobilize sufficient stem cells in a first harvest; therefore, a second stimulation and harvest had to be performed. Eventually, we were able to transplant between 5.6 and 12.3x 10^6^ selected CD34+ stem cells/kg body weight in these three recipients, which were infused together with a T cell add-back of 1x 10^6^ CD3+ T cells/kg body weight (**Table 1**). No signs of graft-versus-host disease (neither acute nor chronic) were seen during the whole follow-up in all three recipients.

**Figure 5:**
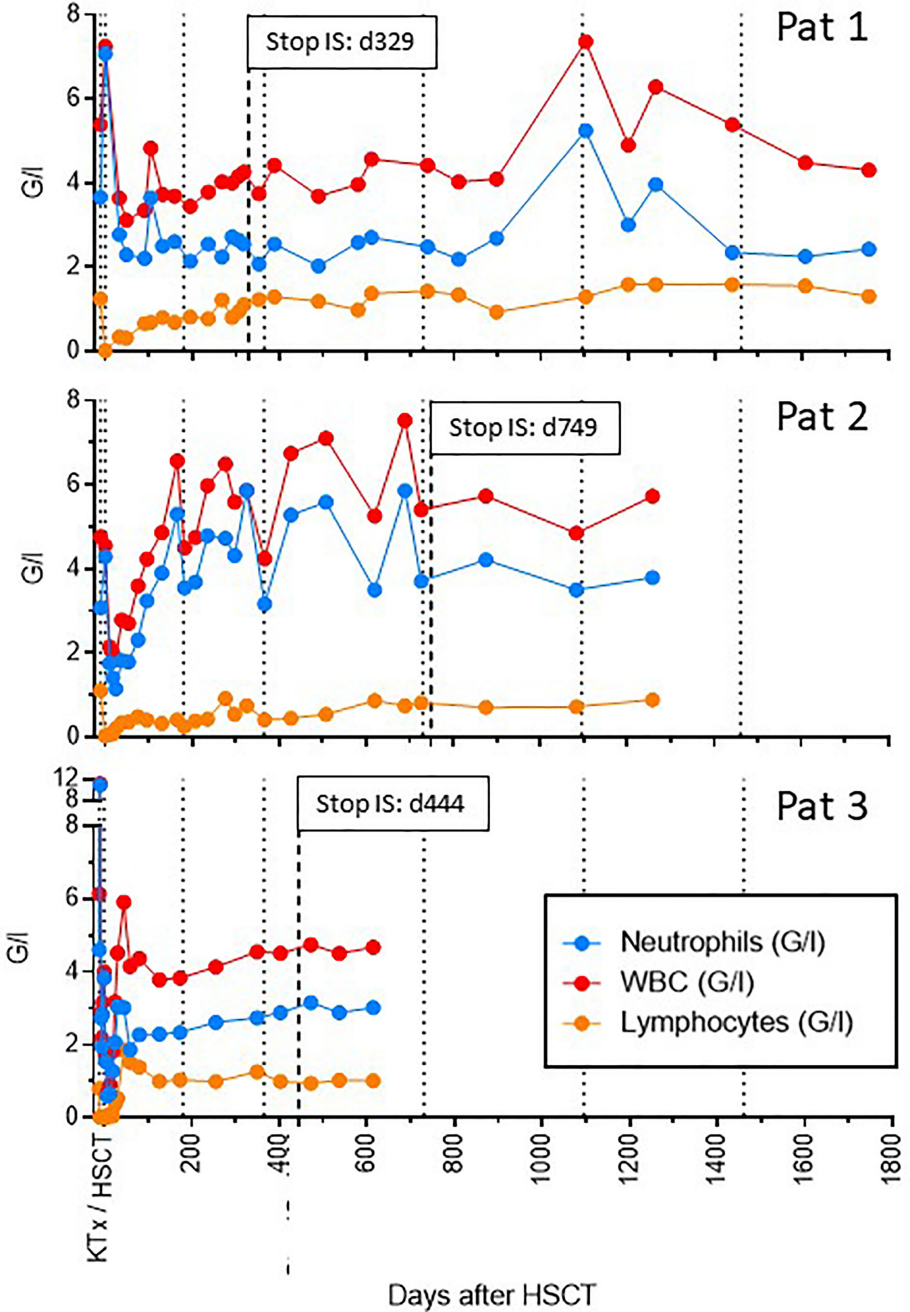
Synopsis of hematological parameters over time. The number of total leukocytes, neutrophils and lymphocytes is shown over time for all three recipients. Dotted lines indicate time intervals of 6 and 12 months post-transplant and yearly thereafter. The hatched line indicates the time point of stop of all immunosuppressive treatment (IS). Profound lymphopenia around the time of transplantation was seen in all three recipients. However, only patient 3 also experienced transient neutropenia, which resolved by 4 weeks post-transplant.

All patients developed as expected a profound lymphopenia around the time of HSCT (**Figure 5**). Hemoglobin and platelet levels remained stable. Only patient 3 developed transient neutropenia (nadir 600 neutrophils/µl) three weeks post-kidney transplant, which however resolved again two weeks later. The other two recipients never experienced neutropenia. We did not observe any severe infectious complications in any of the patients neither early nor late post-transplant (**Table 2**)

**Table 2:** Trial outcome overview.

Patient No		Patient 1	Patient 2	Patient 3
**Kidney transplant**	Primary graft function	Yes	Yes	Yes
	Acute rejection	No	No	No
	Other diagnoses	Yes, biopsy-proven GN	No	Yes, polyomavirus nephropathy
**HSCT**	Chimerism 1 mts	Yes	Yes	Yes
	Chimerism 12 mts	Yes	Yes	Yes
	Chimerism 24 mts	Yes	No	NA
	Acute GvHD	No	No	No
	Chronic GvHD	No	No	No
**Immuno-suppression**	Standard trial immunosuppression until months 6	Yes	Yes	Yes
	Cyclosporine A weaning initiation	Month 7	Month 13	Month 10
	Cyclosporine A Stop	Month 11	Month 25	Month 15
**Complications**	Viral infections	Asymptomatic CMV reactivation	None	BK viremia with nephropathy
	Bacterial infections	Uncomplicated urinary tract infection	None	None
	Non-infectious complications	Calcineurin inhibitor pain syndrome (CIPS)	Gastric ulcer	Lymphocele (→ fenestration)

CMV, cytomegalovirus; GN, glomerulonephritis; GvHD, graft-versus-host disease; HSCT, hematopoietic stem cell transplantation; mts, months; NA, not applicable; No, number.

**Figure 6:**
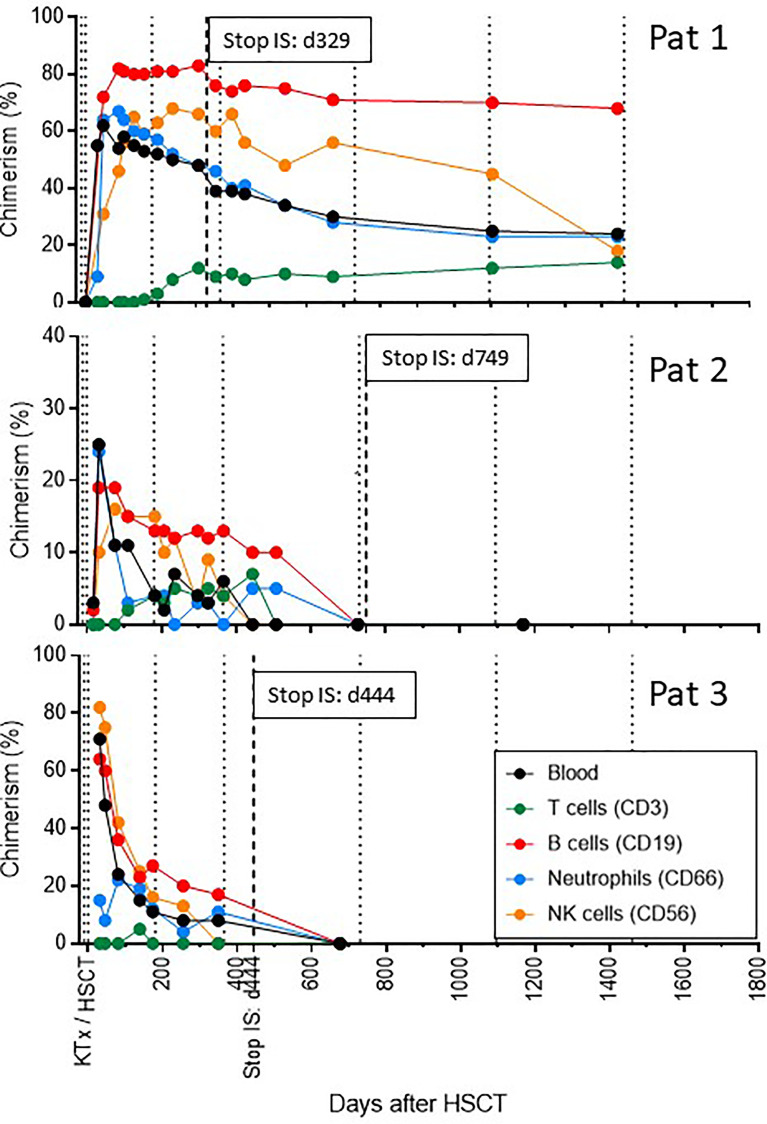
Synopsis of donor chimerism over time. The level of whole blood as well as lineage-specific donor chimerism is shown over time for all three recipients. Dotted lines indicate time intervals of 6 and 12 months post-transplant and yearly thereafter. The hatched line indicates the time point of stop of all immunosuppressive treatment (IS).

The evolution of donor chimerism followed different patterns (**Figure 6**) (9):

- Patient 1 achieved a maximum whole donor blood chimerism of 62%, which then very slowly declined over time, but remained stable between 20-30% until 4 years post-transplant. She was the only recipient who also developed a long-lasting donor T cell chimerism.
- Patient 2 received the lowest number of donor CD34+ cells and experienced the lowest level of whole blood donor chimerism of maximally 25%, which was never stable and slowly declined over time. Therefore, cyclosporine A tapering was started later and delayed in this patient. She finally lost whole blood chimerism by day 500. Immunosuppression was anyway withdrawn by d749, and tolerance is maintained more than 18 months after stop of cyclosporine A.
- Patient 3 achieved the highest levels of whole blood donor chimerism early on (71%), which then rapidly declined and seemed to stabilize on a much lower level. Cyclosporine tapering was therefore started only 10 months post-transplant, and it was stopped 5 months later. This patient remains tolerant 8 months after stopping immunosuppression. This patient never developed substantial T cell chimerism, and lost whole blood chimerism after stop of cyclosporine A.

#### 3.4.3 Specificity of Tolerance and Impact During the COVID-19 Pandemic

Renal transplant recipients have been shown to develop SARS-CoV-2-specific antibodies in only about 50% of cases after application of two vaccine doses. In case of SARS-CoV-2 infection (COVID-19) morbidity and mortality in this patient population is high.

During the COVID-19 pandemic, all three of our trial patients were vaccinated against SARS-CoV-2 with the mRNA vaccine BNT162b2 (Comirnaty^®^, Pfizer/BioNTech). None of the patients suffered from COVID-19. Several months post-vaccination, specific antibody as well as T-cell responses were assessed in all three patients. All three patients developed antibody responses with titers considered to be protective. A SARS-CoV-2-specific T cell response could be detected in 2 out of the three patients. In addition, all of them showed evidence of CMV-specific cellular immunity (**Table 3**).

**Table 3:** SARS-CoV-2-specific vaccination and immunity.

Patient No		Patient 1	Patient 2	Patient 3
**Vaccination**	Vaccine	Comirnaty ^®^	Comirnaty ^®^	Comirnaty ^®^
	1^st^ vaccination	29.1.2021	29.4.2021	14.1.2021
	2^nd^ vaccination	5.3.2021	18.5.2021	11.2.2021
**SARS-CoV-2-specific antibody response**	Elecsys ^®^ (Roche), anti-NP IgG (<1.0)	Not reactive, 0.074	Not reactive, 0.076	Not reactive, 0.075
	Elecsys ^®^ (Roche), anti-Spike IgG (<0.8)	Positive, 1488 U/ml	Positive, 1951 U/ml	Positive, 919 U/ml
	ABCORA ®, (IMV) neutralization score (protective score > 17)	Protective, 28.2	Protective, 83.9	Protective, 40.3
**SARS-CoV-2-specific T-cell response (net stimulation, % CD3+)**	Concanavalin A	42.7%	55.2%	63%
	St. aureus superantigen	66.9%	67.7%	71%
	CMV antigen	(17%)*	(10%)*	76%
	SARS-CoV-2 SP subunit 1	0	27.1%	5%
	SARS-CoV-2 SP subunit 1	1.1%	14.5%	6%

CMV, cytomegalovirus; NP, nucleoprotein; IMV, Institute of Medical Virology (University of Zurich); SARS-CoV-2, new Coronavirus 2; SP, spike protein; St. aureus, Staphylococcus aureus

*These two analyses were performed in a separate assay. Thus, the absolute levels cannot be directly compared to the Concanavalin A/Superantigen response, but the responses are clearly positive.

These vaccine-induced immune responses during the COVID-19 pandemic demonstrated that the immunological tolerance achieved in our trial patients is indeed specific to the donor, while maintaining the ability to mount an effective immune response against viral spike proteins by this novel mRNA vaccine.

## 4 Discussion

The SARS-CoV-2 pandemic confirmed the necessity to continue searching for the holy grail of transplantation medicine: finding solutions to perform solid organ transplants without the need for life-long immunosuppression. In an era of a global pandemic solid organ recipients are particularly vulnerable due to life-long immunosuppression to prevent allograft rejection. Immunosuppressed solid organ recipients (i) suffer more often from common and opportunistic infections; (ii) infectious diseases tend to have a more severe course and worse outcome compared to non-immunosuppressed patients (10); and (iii) immunosuppressed organ recipients respond less well to vaccines (11–13). More than 50 years of research since the seminal experiments on chimerism and tolerance performed by Billingham, Brent, and Medawar in the 1950s (14) were required until the first clinical trials were performed for combined kidney and hematopoietic stem cell transplantation from the same living donor (15). Meanwhile, 3 US centers have established successful programs for such procedures (Stanford, Boston, and Chicago; **Supplementary Table 1**), and up to 100 patients may have benefited so far from these programs to receive an allogeneic kidney without long-term immunosuppression (16–19).

According to our knowledge, swisstolerance.CH is the first European trial applying an established tolerance induction protocol by an independent group. Replicating the clinical protocol required a dedicated interdisciplinary team, but the outcome data are consistent with the results reported by the group of Strober et al. at Stanford University. We demonstrate that this elaborated protocol – despite its complexity – can be replicated by an independent group in another part of the world. The primary endpoint in our study was achieved by the first three patients presented here: acceptance of an HLA-identical allogeneic kidney without long-term immunosuppression, without acute allograft rejection and without graft-versus-host disease. The tolerance to the graft was further demonstrated by a molecular microscope analysis in one of the patients, where the gene expression pattern was indistinguishable from a normal living donor kidney (8).

The benefits of immunosuppression-free allograft acceptance are expected to become particularly important in the long-term, but several observations suggest that our patients have already taken advantage of this. The number of infection-related complications was low, and none of the patients developed post-transplant metabolic disorders. Patient 1 experienced CIPS, which rapidly resolved after stopping cyclosporine A. BK-polyomavirus nephropathy had a very favorable clinical evolution after tapering of immunosuppression in patient 3.

Tolerance induction, in contrast to general immunosuppression, becomes even more attractive in the unique context of the COVID-19 pandemic. All three patients were transplanted before the pandemic reached Europe. None of them suffered from COVID-19. The patients could be vaccinated with the mRNA vaccine BNT162b2, when they were off immunosuppression, and mounted strong and protective neutralizing SARS-CoV-2-specific antibody responses, and 2/3 also specific T cell responses. The number of patients is not sufficient for a conclusive analysis, but the data suggest a better immunological response to the vaccine in comparison to kidney transplant recipients under immunosuppressive therapy (11–13). This peculiar epidemiological setting, with the opportunity to study in patients the immune response to a novel virus, was instrumental to demonstrate the specificity of the immunological tolerance achieved with this protocol, which allowed acceptance of an allogeneic kidney while maintaining fully protective anti-vaccine responses.

Freedom of immunosuppression was only achieved in the second year after transplantation in 2 out of 3 patients. The reason was a slower cyclosporine A tapering than initially planned due to low (patient 2) or rapidly declining (patient 3) whole blood chimerism. This fact reveals one of the limitations of this current protocol: the donor chimerism levels achieved in an individual recipient-donor pair are unpredictable – in terms of absolute levels, stability, and duration (9). When referring to murine experiments, intermediate stable and multilineage chimerism (including T cells) confers the most robust tolerance status (20, 21), and different approaches have been developed to facilitate the induction of stable mixed chimerism (22–24). However, in non-human primate experiments it was demonstrated that also transient chimerism (if present high and long enough) can lead to tolerance towards an allograft, which is maintained beyond the loss of blood chimerism (25). It is considered that non-deletional tolerance mechanisms (such as regulatory cells of any type) and local adaptations within the graft maintain tolerance in these patients (26, 27). Whether this tolerance status is as robust as in stable mixed chimeras is currently unknown.

Our pilot trial also indicates that one major problem of late kidney allograft loss, namely the recurrence of the primary kidney disease, cannot be solved by the induction of mixed chimerism, at least in the HLA-identical setting (patient 1). The non-myeloablative, minimal intensity protocol for HSCT does not allow for a complete reset of the immune system, and autoantigen presentation will also not be impaired, if the donor displays an identical set of HLA molecules. However, individual case reports indicate that HLA-mismatched HSCT may help improving some primary glomerulopathies, such as IgA nephropathy (28).

In conclusion, by replicating the Stanford protocol we confirm that donor-specific immune tolerance can be achieved in selected patients by mixed hematopoietic chimerism. The first three patients enrolled in our tolerance program were successfully withdrawn from all immunosuppression while maintaining stable allograft function and without signs of rejection or GvHD. Immunocompetence was demonstrated by protective immune responses against the SARS-CoV-2 vaccine in all three patients. The main challenge for the future will be the further development of this protocol across HLA barriers.

## Data Availability Statement

The raw data supporting the conclusions of this article will be made available by the authors, without undue reservation.

## Ethics Statement

The studies involving human participants were reviewed and approved by Ethikkommission der Kantons Zürich, Schweiz. The patients/participants provided their written informed consent to participate in this study.

## Author Contributions

TF, JS, US, and PC designed the trial. TF and PC wrote the ethical board applications and applied for funding. TF, PC, KH, and US wrote the manuscript. KH, OdR, and TM cared for the patients. TG, MH, and US were responsible for preparation of cellular transplant products and for stem cell transplantation. CL and OR were responsible for total lymphoid irradiation procedures. AG and BH analyzed allograft biopsy specimens. JN and IA performed immunological analyses. All authors contributed to the article and approved the submitted version.

## Funding

The Horton Foundation Switzerland (a non-commercial private foundation supporting clinical and basic research) supports this trial.

## Conflict of Interest

The authors declare that the research was conducted in the absence of any commercial or financial relationships that could be construed as a potential conflict of interest.

## Publisher’s Note

All claims expressed in this article are solely those of the authors and do not necessarily represent those of their affiliated organizations, or those of the publisher, the editors and the reviewers. Any product that may be evaluated in this article, or claim that may be made by its manufacturer, is not guaranteed or endorsed by the publisher.
