# Supplementary material for: Successful Induction of Specific Immunological Tolerance by Combined Kidney and Hematopoietic Stem Cell Transplantation in HLA-Identical Siblings
Source: Front Immunol. 2022 Jan 31;13:796456. doi: 10.3389/fimmu.2022.796456 (PMC8841472; doi:10.3389/fimmu.2022.796456)
Supplement: Supplementary file 1 [file DataSheet_1.docx]

Successful induction of specific immunological tolerance by combined kidney and hematopoietic stem cell transplantation

Thomas Fehr ^1,2,†,*^, Kerstin Hübel ^1,3,†^, Olivier de Rougemont ^3^, Irene Abela ^4^, Ariana Gaspert ^5^, Tayfun Güngör ^6^, Mathias Hauri ^6^, Birgit Helmchen ^5^, Claudia Linsenmeier ^7^, Thomas Müller ^1^, Jakob Nilsson ^8^, Oliver Riesterer ^7^, John D. Scandling ^9^, Urs Schanz ^10,‡^, and Pietro Cippà ^1,11,‡^

^1^ Division of Nephrology, University Hospital Zurich, Zurich, Switzerland

^2^ Department of Internal Medicine, Cantonal Hospital Graubuenden, Chur, Switzerland

^3^ Department of Surgery and Transplantation, University Hospital Zurich, Zurich, Switzerland

^4^ Institute of Medical Virology, University of Zurich, Zurich, Switzerland

^5^ Department of Pathology, University Hospital Zurich, Zurich, Switzerland

^6^ Division of Stem Cell Transplantation, University Children's Hospital Zurich – Eleonore Foundation & Children`s Research Center (CRC), Zurich, Switzerland

^7^ Department of Radiation Oncology, University Hospital Zurich, Zurich, Switzerland

^8^ Laboratory for Transplantation Immunology, University Hospital Zurich, Zurich, Switzerland

^9^ Division of Nephrology, Stanford University School of Medicine, Stanford, CA, USA

^10^ Department of Medical Oncology and Hematology, University Hospital Zurich, Zurich, Switzerland

^11^ Division of Nephrology, Ente Ospedaliero Cantonale, Lugano, Switzerland

† These authors have contributed equally and share first authorship.
‡ These authors have contributed equally and share last authorship.

*** Correspondence:**Thomas Fehr
[thomas.fehr@uzh.ch](mailto:thomas.fehr@uzh.ch)

# Supplementary Table 1 – Synoptic presentation of published mixed chimerism protocols

| \| **Transplant Center** \|  \| **Boston** \| **Stanford** \| **Chicago** \|  \|  \| \| --- \| --- \| --- \| --- \| --- \| --- \| --- \| \| **HLA match** \|  \| HLA-matched &  HLA-mismatched \| Established for HLA-matched \| HLA-matched &  HLA-mismatched \|  \|  \| \| **Conditioning regimen** \| Timing \| Pre-transplant \| Post-transplant \| Pre-transplant \|  \|  \| \| Irradiation \| Thymic irradiation \| Total lymphoid irradiation \| Total body  irradiation \|  \|  \| \| Chemo-therapy \| Cyclophosphamide \| None \| Fludarabine, cyclophosphamide \|  \|  \| \| T cell depletion \| Siplizumab  (anti-CD2) \| Anti-thymocyte globulin (ATG) \| None \|  \|  \| \| B cell depletion \| Rituximab (for HLA-mismatched only) \| None \| None \|  \|  \| \| **Cellular transplant** \| Type of transplant \| Bone marrow  (un-manipulated) \| Mobilized peripheral stem cells (T-cell depleted, with controlled T-cell add-back) \| Mobilized peripheral stem cells + “facilitating cells” \|  \|  \| \| Chimerism achieved \| Mixed; mostly transient \| Mixed; some transient, some long-term \| Full donor in majority \|  \|  \| \| **Main outcome** \| Acute rejection \| No \| No \| No \|  \|  \| \| SIRS \| High risk \| Low risk \| Low risk \|  \|  \| \| GvHD \| No \| No \| Yes \|  \|  \| \| Recurrent disease \| Yes \| Yes \| Rare \|  \|  \| |
| --- | --- | --- | --- | --- | --- | --- | --- | --- | --- | --- | --- | --- | --- | --- | --- | --- | --- | --- | --- | --- | --- | --- | --- | --- | --- | --- | --- | --- | --- | --- | --- | --- | --- | --- | --- | --- | --- | --- | --- | --- | --- | --- | --- | --- | --- | --- | --- | --- | --- | --- | --- | --- | --- | --- | --- | --- | --- | --- | --- | --- | --- | --- | --- | --- | --- | --- | --- | --- | --- | --- | --- | --- | --- | --- | --- | --- | --- | --- | --- | --- | --- | --- | --- |

*Abbreviations:* DC, dendritic cells; GvHD, graft-versus-host disease; NK, natural killer cells; SIRS, systemic inflammatory response syndrome

# Supplementary Table 2 – Banff classification of kidney biopsies

| Patient | Biopsy No / date | Banff classification |
| --- | --- | --- |
| Patient 1 | 1/ time0 biopsy | t0,i0,ti0, ptc0,v0,cv0,g0,cg0, mm0,ci1,ct1,ah1,aah0,i-IFTA0, C4d na |
|  | 2/ 6 months | t1,i0,ti0, ptc0,v0,cv0,g0,cg0, mm0,ci0,ct1,ah1,aah2,i-IFTA0, C4d0 |
|  | 3/ 11 months * | t0,i0,ti0, ptc0,v0,cv0,g1,cg1b, mm0,ci0,ct1,ah1,aah0,i-IFTA0, C4d0 |
|  | 4/ 25 months * | t0,i0,ti0, ptc0,v0,cv0,g na, cg na, mm0,ci1,ct1,ah0,aah0,i-IFTA0, C4d0 |
| Patient 2 | 1/ 6 months | t0,i0,ti0, ptc0,v0,cv0,g0,cg0, mm0,ci1,ct1,ah0,aah0,i-IFTA0, C4d0 |
|  | 2/ 12 months | t0,i0,ti0, ptc0,v0,cv1,g0,cg0, mm0,ci1,ct1,ah0,aah0,i-IFTA0, C4d0 |
|  | 3/ 18 months | t0,i0,ti0, ptc0,v0,cv1,g1,cg1b, mm0,ci0,ct1,ah1,aah0,i-IFTA0, C4d0 |
|  | 4/ 24 months | t0,i0,ti0, ptc0,v0,cv2,g0,cg0, mm0,ci0,ct1,ah1,aah0,i-IFTA2, C4d0 |
| Patient 3 | 1/ 6 months | t0,i0,ti0, ptc0,v0,cv1,g0,cg0, mm0,ci0,ct0,ah1,aah0,i-IFTA0, C4d0 |
|  | 2/ 12 months ** | t2,i1,ti1, ptc0,v0,cv0,g0,cg0, mm0,ci0,ct0,ah1,aah0,i-IFTA3, C4d0, pv2 |

na not assessed
* A diagnosis of C3-glomerulonephritis (de novo or recurrent) was made.
** A diagnosis of BK polyomavirus nephropathy was made, which explains the tubulointerstitial
 findings.
