# Supplementary material for: Successful Induction of Specific Immunological Tolerance by Combined Kidney and Hematopoietic Stem Cell Transplantation in HLA-Identical Siblings
Source: Front Immunol. 2022 Jan 31;13:796456. doi: 10.3389/fimmu.2022.796456 (PMC8841472; doi:10.3389/fimmu.2022.796456)
Supplement: Supplementary file 2 [file Presentation_1.pptx]

## Slide 1
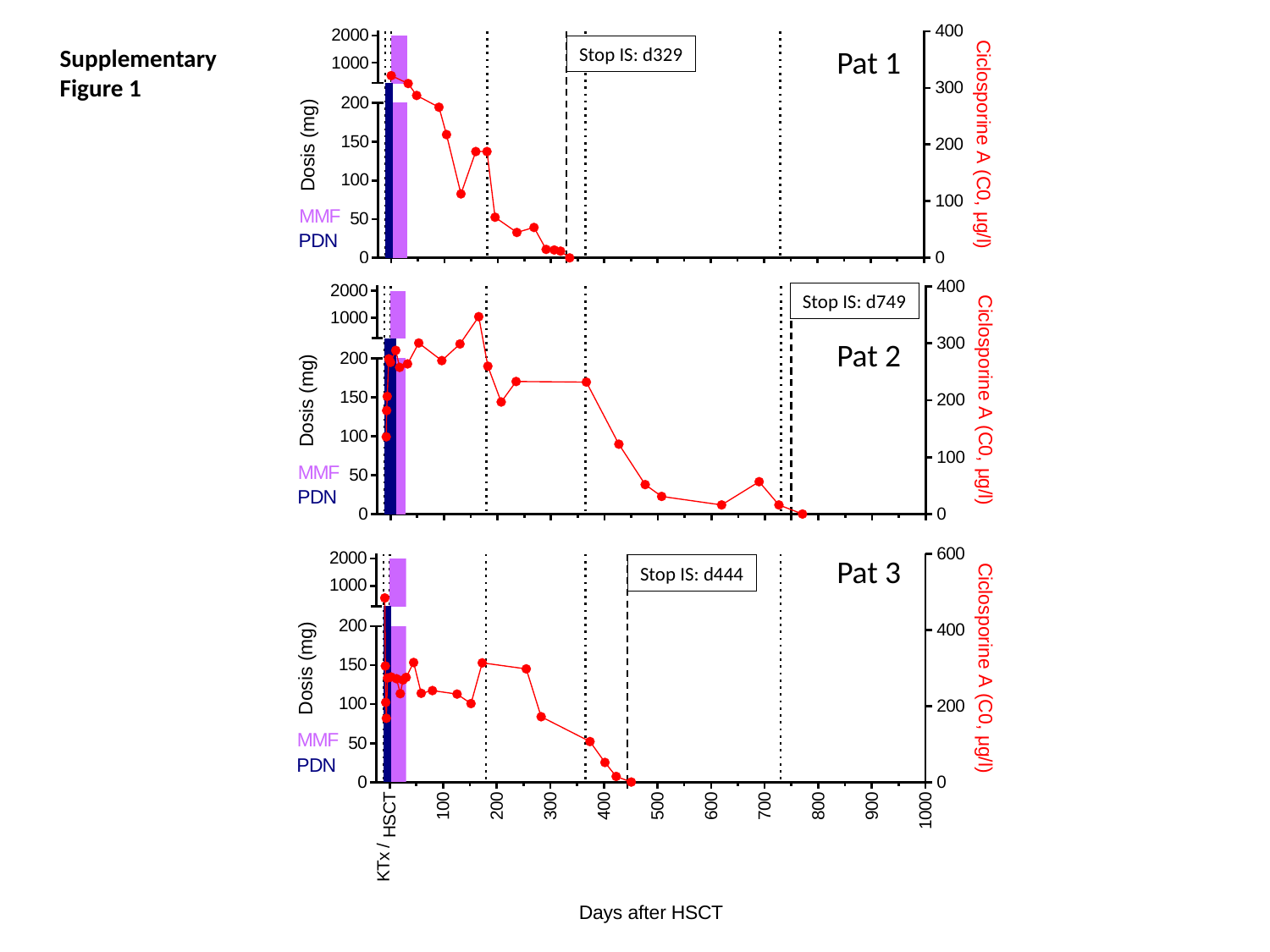

Supplementary
Figure 1
Stop IS: d329
Pat 1
Stop IS: d749
Pat 2
Pat 3
Stop IS: d444

## Slide 2
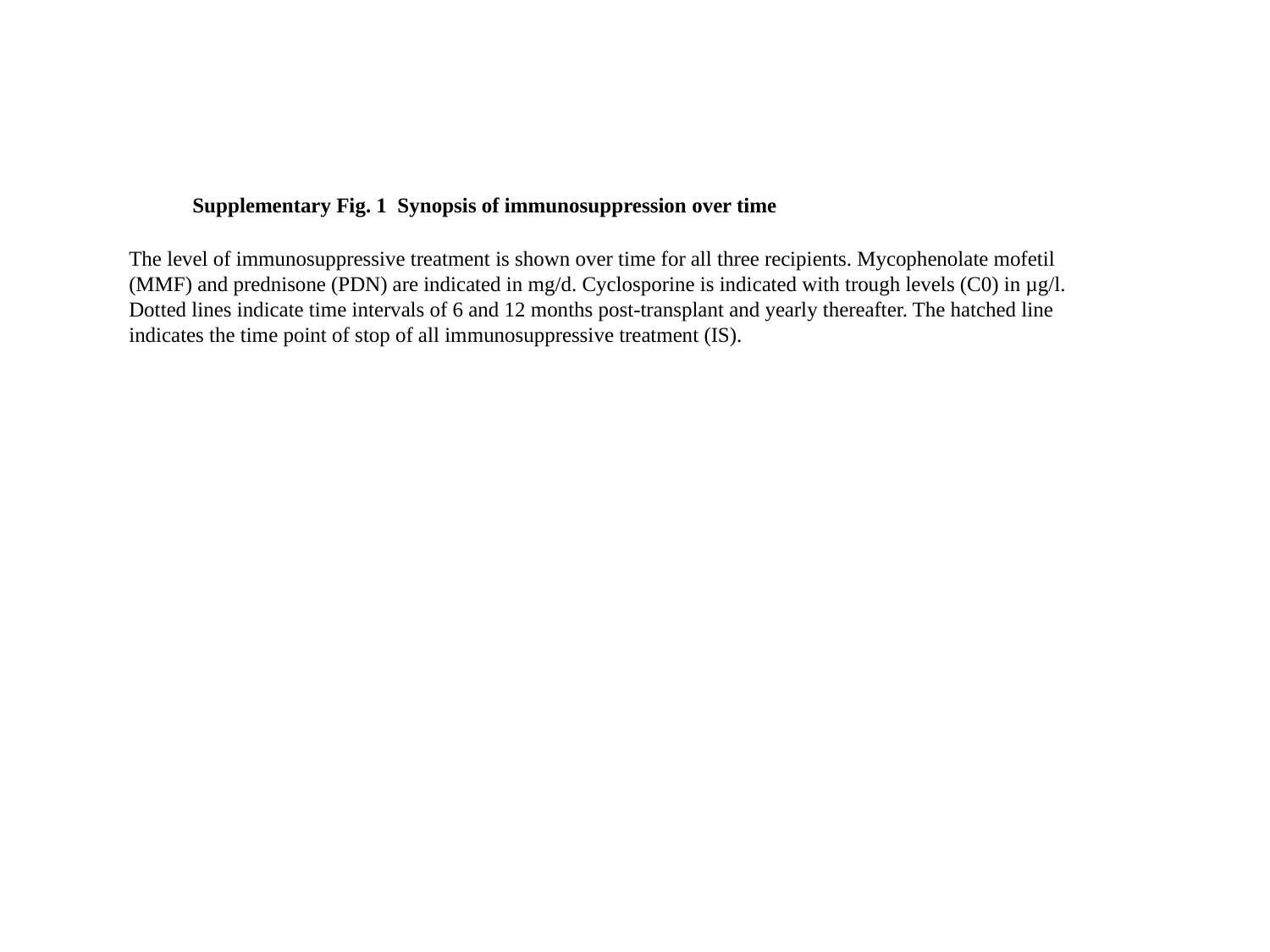

Supplementary Fig. 1 Synopsis of immunosuppression over time
The level of immunosuppressive treatment is shown over time for all three recipients. Mycophenolate mofetil (MMF) and prednisone (PDN) are indicated in mg/d. Cyclosporine is indicated with trough levels (C0) in µg/l. Dotted lines indicate time intervals of 6 and 12 months post-transplant and yearly thereafter. The hatched line indicates the time point of stop of all immunosuppressive treatment (IS).
